# Predictors of withdrawal of anticancer drug indications granted accelerated approval: a retrospective cohort study

**DOI:** 10.1016/j.eclinm.2025.103088

**Published:** 2025-05-31

**Authors:** Ariadna Tibau, Edward R. Scheffer Cliff, Alejandra Romano, Maria Borrell, Consolacion Molto, Aaron S. Kesselheim

**Affiliations:** a Program on Regulation, Therapeutics, And Law (PORTAL), Division of Pharmacoepidemiology and Pharmacoeconomics, Department of Medicine, Brigham and Women's Hospital, Boston, Massachusetts, USA; b Harvard Medical School, Boston, Massachusetts, USA; c Oncology Department, Hospital de la Santa Creu i Sant Pau, Institut d’Investigació Biomèdica Sant Pau, Barcelona, Catalonia, Spain; d Department of Medicine, Universitat Autònoma de Barcelona, Barcelona, Catalonia, Spain; e Department of Clinical Haematology, Peter MacCallum Cancer Centre and Royal Melbourne Hospital, Melbourne, Australia; f Sir Peter MacCallum Department of Oncology, The University of Melbourne, Melbourne, Australia; g Vall d'Hebron Institute of Oncology (VHIO), Barcelona, Catalonia, Spain; h Medical Oncology Department, Vall d'Hebron Hospital, Barcelona, Catalonia, Spain; i R.S. McLaughlin Durham Regional Cancer Centre, Oshawa, Ontario, Canada; j Department of Oncology, Queen's University, Kingston, Ontario, Canada; k Division of Cancer Care and Epidemiology, Queen's Cancer Research Institute, Kingston, Ontario, Canada

**Keywords:** Accelerated approval, Drug withdrawal, Food and drug administration (FDA), European Society of Medical Oncology-Magnitude of Clinical Benefit Scale (ESMO-MCBS), Cancer therapy

## Abstract

**Background:**

The accelerated approval pathway allows the FDA to approve drugs for serious conditions based on promising surrogate measures, with confirmatory studies required later. If subsequent testing shows an unfavorable benefit-risk profile, the indication may be withdrawn. This study aimed to identify factors associated with the withdrawal of drug indications following accelerated approval.

**Methods:**

In this retrospective cohort study, we identified FDA-approved drugs for solid and hematologic cancers from 1992 to 2022 and extracted factors present at the time of accelerated approval, including pivotal trial characteristics, outcomes, and confirmatory study initiation timing from drug labels and published reports. Clinical benefit was assessed using the European Society of Medical Oncology-Magnitude of Clinical Benefit Scale (ESMO-MCBS), with high benefit as A-B/4–5 and low as C/≤2. Multivariable logistic regression identified factors associated with drug indication withdrawal.

**Findings:**

Among 167 accelerated approval indications for 113 anticancer drugs, by August 2024, 102 (61%) had been converted to regular approval, 31 (19%) were withdrawn, and the remaining 34 (20%) were ongoing accelerated approvals. Of the 133 indications that were either converted or withdrawn, 52 (39%) were approvals for hematologic cancer drugs, and 41 (31%) supported genome-targeted drug approvals. Among 83 eligible indications, 46 (55%) were granted Breakthrough Therapy designation. In the 133 pivotal trials, 112 (84%) used response rate as the primary endpoint, and 66% (86/130) offered low clinical benefit on the ESMO-MCBS. In multivariable analysis, Breakthrough Therapy designations (OR 0.26; 95% CI, 0.10–0.75; p = 0.01) and indications for genome-targeted therapies (OR 0.26; 95% CI, 0.08–0.80; p = 0.02) were associated with lower withdrawal rates. Higher withdrawal rates were associated with low ESMO-MCBS scores (OR, 4.63; 95% CI, 1.50–14.33; p = 0.008).

**Interpretation:**

Accelerated approvals based on early data suggesting limited clinical benefit tend to have higher withdrawal rates, whereas therapies with Breakthrough Therapy designation and genome-targeted mechanisms are more likely to validate clinical benefits and achieve regular approval. Patients and healthcare providers should consider these factors when evaluating whether to use therapies granted accelerated approval.

**Funding:**

10.13039/100008052Alfonso Martín Escudero Foundation (to AT) and Arnold Ventures, the Commonwealth Fund, and Kaiser Permanente Institute for Health Policy Research (to ASK).

**Research in context**

**Evidence before this study**

The FDA's accelerated approval pathway has expedited access to cancer treatments by allowing approvals based on surrogate measures, contingent upon the completion of confirmatory trials that are supposed to validate definitive clinical benefits, such as overall survival or improvements in quality of life. This program has facilitated the introduction of some transformative therapies, but concerns remain about the number of withdrawn accelerated approvals and delays in the withdrawal of drugs without demonstrated benefits. As of August 2024, 200 oncology indications had received accelerated approval, with 105 subsequently confirmed by the FDA and 31 withdrawn, with the vast majority of withdrawals occurring in the last five years. Given that approximately one in five approvals in the past decade have been withdrawn, we sought to identify the factors associated with withdrawal. A PubMed search conducted up to August 2024, using terms such as “accelerated approval,” “oncology,” and “drug withdrawal,” revealed no previous studies systematically predicting withdrawal. This study addresses this gap by analyzing pivotal trial characteristics, clinical benefit assessments, and the status of confirmatory trials at the time of approval to identify predictors of withdrawal.

**Added value of this study**

This study offers a comprehensive analysis of the factors influencing the withdrawal of cancer drug indications approved through the accelerated approval pathway from 1992 to 2022. Among the 133 accelerated approval indications examined that were later converted or withdrawn, we found that those based on pivotal trials demonstrating low clinical benefit—assessed using validated scales like the ESMO-MCBS—were more likely to be withdrawn. By contrast, Breakthrough Therapy designations and indications supporting genomic-targeted therapies were associated with reduced risk of withdrawal. Over the past decade, these factors have effectively identified therapies that are likely to demonstrate genuine clinical benefit, highlighting the evolving criteria influencing drug withdrawals in oncology.

**Implications of all the available evidence**

Accelerated approvals based on early data indicating substantial clinical benefit are more likely to confirm those benefits and achieve regular approval. In March 2023, the FDA released draft guidance to improve clinical trial design for cancer therapies seeking accelerated approval, emphasizing the assessment of “clinical meaningfulness” alongside statistical significance, although its definition remains unclear. Our findings support using value frameworks to identify drugs with low or uncertain clinical benefits, aiding decision-making for patients and regulators. Drugs with clear mechanisms of action targeting specific patient groups—such as genome-targeted therapies—should be prioritized for accelerated approval. Similarly, therapies showing meaningful improvements over existing treatments merit prioritization.

These insights can help regulators refine the selection process and provide valuable guidance to patients and healthcare providers. Further work is needed to enhance the evaluation framework, ensuring alignment with emerging clinical evidence.

## Introduction

The FDA's accelerated approval pathway is frequently used to expedite the approval of new oncology therapies for patients with an unmet medical need. Accelerated approvals are based on changes to surrogate measures—such as cancer response rate or radiologic appearance—that the FDA deems “reasonably likely to predict a clinical benefit” and are contingent on the drug's sponsor conducting confirmatory studies to verify that the drug indeed provides a clinical benefit.

Over the years, the FDA's accelerated approval program has facilitated the introduction of transformative cancer treatments, which have become essential to contemporary oncology.^1^ Notable examples include fam-trastuzumab deruxtecan-nxki for *HER2*-positive breast cancer^2^ and pembrolizumab for metastatic melanoma^3^ and non-small cell lung cancer.^4^ However, recent scrutiny of the program highlights concerns over reliance of confirmatory trials still evaluating surrogate measures rather than definitive clinical outcomes,^5^ delays in the completion of confirmatory trials,^6^ and the slow withdrawal of indications when trials fail to confirm clinical benefit.^7^

While accelerated approval ensures early access to drugs for patients, the uncertainty of clinical benefit can lead to the widespread use of drugs later shown to have an unfavorable benefit-risk profile for the specific indication, which may later be withdrawn.^8^ The process for withdrawing indications if confirmatory trials fail to meet the protocol-specified primary endpoint or demonstrate clinical benefit has proven to be cumbersome, with such indications remaining on the market for months or even years before being revoked.^9^^,^^10^ As of August 2024, of 200 oncology accelerated approvals to date,^11^ 105 have had confirmatory studies leading to conversion,^12^ while 31 oncology accelerated approval indications have been withdrawn,^13^ with the most recent 24 withdrawals concentrated in just the last 5 years.

Given that approximately one in five oncology accelerated approvals in the last decade have been withdrawn from the US market, this study focuses on identifying factors present at the time of accelerated approval that are associated with the subsequent withdrawal of anticancer indications. By analyzing regulatory pathways and pivotal trial characteristics, clinical benefit assessments, and the initiation of confirmatory studies, we seek for the first time to our knowledge to identify key predictors of withdrawal, which can impact the way that these drugs are used in practice.

## Methods

### Data sources

From the FDA website, we identified cancer drugs that received accelerated approval from the inception of the program in 1992 through 2022.^14^ We further reviewed the status of confirmatory trials through August 30, 2024 to identify which indications had been withdrawn^13^ or converted to regular approval.^12^

### Data extraction

We gathered information on the basis for accelerated approvals, including expedited regulatory pathways such as priority review,^15^^,^^16^ Breakthrough Therapy designation,^17^ and Orphan Drug Act designation.^18^ We documented the dates of accelerated approval, and any subsequent conversions to regular approval or withdrawals. To assess study quality, we reviewed key methodological features of the pivotal clinical trials supporting accelerated approval. These included the number of pivotal trials, sample sizes, trial designs (randomized vs. single-arm), trial phases (phase 1-2 vs. phase 3), and primary endpoints leading to approval (overall survival vs. time-to-event intermediate endpoints vs. non-time-to-event intermediate endpoints). When multiple pivotal trials supported a single accelerated indication, we prioritized those with the most robust endpoints, giving precedence to overall survival over time-to-event intermediate endpoints, and the latter over non-time-to-event endpoints. If the studies shared the same approval-supporting endpoint, the one with the best results was selected. For example, in cases when multiple single-arm trials reported response rate as the only endpoint, we prioritized the trial with the highest response rate. We also recorded drug information, including the therapy type (cytotoxic, immune checkpoint inhibitors, or targeted therapies), and identified genome-targeted drugs, defined by their approval based on a genomic test.^19^ This focus reflects the FDA's impact on advancing precision oncology through accelerated approval of targeted therapies.^20^ For withdrawn drugs, we collected the reasons for their removal.

We searched ClinicalTrials.gov to verify the status of confirmatory studies at the time of accelerated approval, as prior studies have identified them as a key factor influencing drug withdrawal.^21^ Our findings were cross-referenced with an FDA-published list to ensure accuracy.^22^

For this analysis, all characteristics were assessed at the time of accelerated approval, prior to market authorization.

### Data synthesis and scoring

Using FDA drug labels^23^ and clinical trial reports, we assessed the pivotal trials with the European Society of Medical Oncology-Magnitude of Clinical Benefit Scale (ESMO-MCBS) version 1.1 for solid cancers^24^ and ESMO-MCBS version 1.0 for hematological malignancies.^25^ When available, we cross-referenced our results with the ESMO-MCBS scorecards for solid tumor trials^26^ and the ESCM-MCBS-H appendix for hematological malignancies.^25^ Substantial clinical benefit was defined as a grade of A or B for curative trials and grades 4 or 5 for palliative trials.^24^ Intermediate benefit was categorized as grade 3, while low benefit was defined as grades 1, 2, or C.^27^ Supplementary Table S1 details the available forms, grades of evidence, and representative examples.

Sample identification, data extraction, and data scoring were independently performed by two investigators (CM and MB or AR and AT). Any discrepancies were resolved through discussion.

### Statistical analysis

Data were summarized using proportions, medians, and interquartile ranges. Before applying non-parametric methods, we assessed the normality of the data using graphical methods (such as histograms) and the Shapiro–Wilk test. For data that did not meet the normality assumption, we applied non-parametric methods for analysis. Logistic regression was used to identify factors associated with drug indication withdrawal. Univariate analyses were conducted first, followed by multivariable analyses. Initially, three multivariable models were constructed, each incorporating all variables of interest, irrespective of statistical significance. These included (1) expedited approval pathways and confirmatory study status at the time of accelerated approval, (2) therapeutic drug types, and (3) clinical trial characteristics, including the ESMO-MCBS clinical benefit scale. To prevent overfitting, variable selection was based on ensuring appropriate model fit, limiting one variable per 10 events. As a second step, a sensitivity analysis was performed by repeating the multivariable logistic regression, this time including only variables that were significant in the univariate analysis. Results were reported as odds ratios (OR) with 95% confidence intervals (CI).

Finally, given the significant advances in cancer treatment over the past decade, largely driven by the introduction of immune checkpoint inhibitors, antibody-drug conjugates, and genome-targeted therapies, we also conducted an exploratory multivariable sensitivity analysis, distinguishing the most recent decade (2013–2022) from the prior two decades (1992–2012) and an analysis was also performed excluding the last five years (1992–2017).

All p-values were evaluated two-sided. A significance level of p < 0.05 was initially established; however, the Bonferroni correction was applied to adjust for multiple comparisons, leading to a more stringent adjusted significance threshold. Statistical analyses were performed using SPSS (version 28.0 for Windows). The study followed the STROBE guidelines for cohort studies.

The study was considered exempt from ethical approval as it used non-identifiable data and did not involve research with human participants.

### Role of funding

The funders of the study had no role in its design, data collection, analysis, interpretation, or writing. All authors had complete access to the study data, and the corresponding author had the final responsibility for the decision to submit for publication.

## Results

**Fig. 1:**
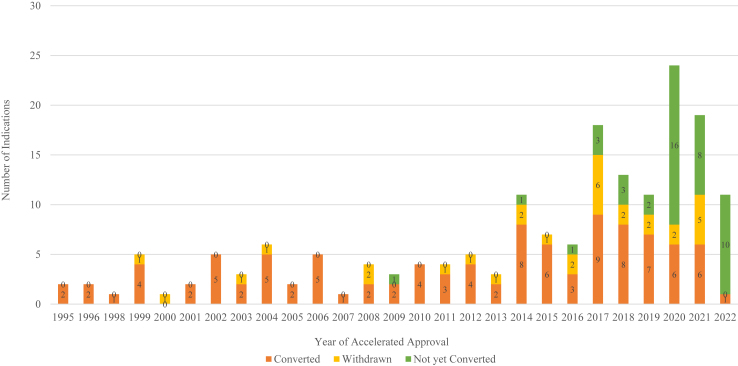
**Accelerated approval of malignant hematology and oncology drugs and biologics converted to regular approval or withdrawn by year, 1992–2022**. The graph shows the number of malignant hematology and oncology drug and biologic indications that received accelerated approval from 1992 to 2022, categorized by their subsequent status: converted to regular approval (orange), withdrawn (yellow), or not yet converted (green).

Between 1992 and 2022, the FDA granted accelerated approval to 113 anticancer drugs covering 167 new cancer indications. As of August 2024, 102 (61%) had been converted to full approval (Supplementary Table S2), 31 (19%) had been withdrawn (Supplementary Table S3), and 34 (20%) remain ongoing. Among the accelerated approval indications with either verified clinical benefit or lack thereof, 31 out of 133 (23%) have been withdrawn (Fig. 1). The median timeframe for the conversion from accelerated to regular approval was 3.10 years (IQR: 1.90–4.83 years), compared to 3.83 years (IQR: 2.83–7.58 years) for the median time to withdrawal after accelerated approval. The overall median observation period for the study was 3.29 years (IQR: 2.20–5.31 years), covering the time from accelerated approval to withdrawal or conversion to full approval.

### Indications for accelerated approval and characteristics of pivotal trials

Key characteristics of the indications and pivotal trials are detailed in Table 1. Of the 133 accelerated approvals either converted or withdrawn, 80 (60%) were for initial indications. The pathway was associated with Orphan Drug Act designation in 86 (65%) cases, Priority Review in 107 (80%), and Breakthrough Therapy designation in 46 of 83 (55%) cases.

**Table 1:** Characteristics of indications and clinical trials for oncology drug indications granted accelerated approval (1992–2022) and subsequently converted or withdrawn.

Characteristics	No. of indications and pivotal trials (%)
	133 (100)
Subsequent regulatory outcome	
Regular approval	102 (77)
Withdrawn	31 (23)
Indication types	
New molecular entities	80 (60)
Supplemental indications	53 (40)
Orphan Drug Act designation	
Yes	86 (65)
No	47 (35)
Priority review	
Yes	107 (80)
No	26 (20)
Breakthrough Therapy Designation^a^	
Yes	46/83 (55)
No	37/83 (45)
Number of trials supporting approval	
1	104 (78)
>1	29 (22)
Sample size	
Median	130 patients
IQR	86.5–216.5 patients
Tumor type	
Solid cancer	81 (61)
Hematologic cancer	52 (39)
Mechanism of action	
Kinase inhibitor	56 (42)
Immune checkpoint inhibitor	29 (22)
Cytotoxic chemotherapy	18 (14)
Endocrine therapy	4 (3)
Therapeutic antibody	15 (11)
Antibody drug conjugate	11 (8)
Genome-targeted therapies	
Yes	41 (31)
No	92 (69)
Confirmatory trial commenced at time of accelerated approval	
Yes	100 (75)
No	33 (25)
Pivotal trial study design	
Randomized	43 (32)
Single-arm	90 (68)
Phase of study	
I–II	106 (80)
III	27 (20)
Blinding^b^	
Open-label	33/43 (77)
Double-blind	10/43 (23)
Primary endpoint	
Overall survival	2 (2)
Intermediate endpoint	131 (98)
Response Rate	112
Progression-free survival	13
Disease-free survival	5
Reduction in the number of colorectal polyps	1
ESMO-MCBS^c^	
High clinical benefit (grades 4-5/A-B)	13/130 (10)
Intermediate clinical benefit (grade 3)	31/130 (24)
Low clinical benefit (grades 1-2/C)	86/130 (66)

Abbreviations: ESMO-MCBS, European Society for Medical Oncology Magnitude of Clinical Benefit; IQR, Interquartile Range.

a Breakthrough therapy designation came into effect in July 2012.

b Blinding is only calculated among randomized trials.

c Three trials supporting accelerated approval were deemed unscorable within the ESMO-MCBS framework: bicalutamide (accelerate approval date 4-Oct-1995, for advanced prostate cancer), celecoxib (accelerate approval date 23-Dec-99, for reducing the number of adenomatous colorectal polyps in familial adenomatous polyposis patients), and tositumomab (accelerate approval date 22-Dec-04, for relapsed or refractory low-grade follicular not treated with rituximab).

Among the accelerated approved indications, 52 (39%) were granted for malignant hematology and 81 (61%) for solid tumor drugs. Forty-one approvals (31%) were for genome-targeted therapies and 29 (22%) for Immune Checkpoint Inhibitor.

The 133 indications that received accelerated approval were supported by 133 pivotal trials. Ninety (68%) were single-arm trials, 106 (80%) were Phase I/II trials, and 33 of the 43 randomized trials (77%) were open-label. Of the trials, 112 (84%) used response rate as the primary endpoint. By contrast, 13 (10%) used progression-free survival, 5 (4%) employed disease-free survival, 1 (1%) focused on the reduction in the number of polyps, and 2 (1%) evaluated overall survival as the primary endpoint. The median sample size was 130 patients (IQR: 86.5–216.5).

Thirty-three confirmatory trials (25%) were not underway at the time of accelerated approval. For indications eventually withdrawn, the median time to withdrawal was 3.81 years (IQR 2.83–6.33) for drugs with ongoing confirmatory trials at the time of accelerated approval, compared to 7.31 years (IQR 2.73–9.84) for those without active confirmatory trials.

Reasons for withdrawal are detailed in Supplementary Table S3: lack of benefit (12 cases, 39%), absence of confirmatory clinical data (9 cases, 29%), and safety and/or efficacy concerns (10 cases, 32%).

### ESMO-MCBS thresholds

The ESMO-MCBS framework was applicable to 130 of the 133 (98%) pivotal trials that supported accelerated approval. Of these 130 trials, 13 (10%) met the ESMO-MCBS threshold for substantial clinical benefit, 31 (24%) demonstrated an intermediate level of clinical benefit, and 86 (66%) provided low benefit. Discrepancies were identified in 25% of the scored cases and resolved through investigator discussion.

### Associations with verified benefit or withdrawal

**Fig. 2:**
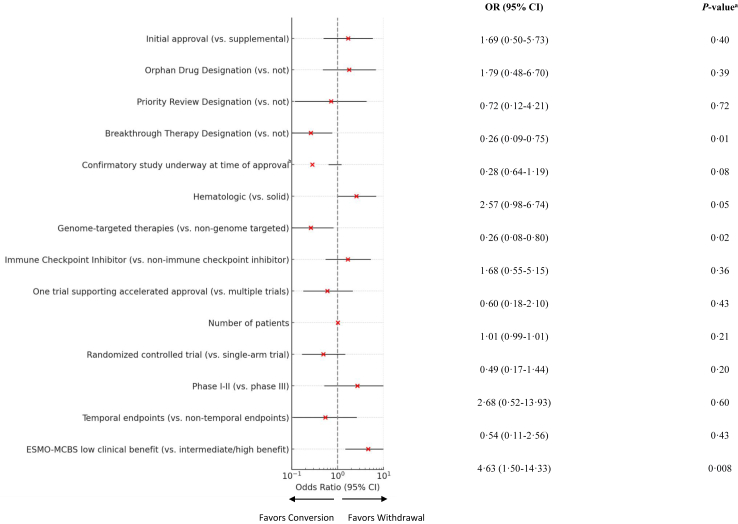
**Forest Plot of characteristics associated with withdrawn indications in multivariable logistic regression models**. This forest plot illustrates the odds ratios (ORs) and 95% confidence intervals (CIs) for variables associated with the withdrawal of indications granted accelerated approval. Results are based on three multivariable logistic regression models, each analyzing distinct sets of variables: (1) expedited approval pathways and confirmatory trial status at the time of accelerated approval, (2) therapeutic drug types, and (3) clinical trial characteristics, including the ESMO-MCBS clinical benefit scale. The analysis includes 133 accelerated approvals. Notes: a. p values are two-sided. Statistical significance was set at p < 0.05 and adjusted using Bonferroni correction for multiple comparisons. b. Indications with confirmatory trials commenced prior to the time of accelerated approval initially showed lower odds of withdrawal (OR 0.21; 95% CI, 0.09–0.81; p = 0.02); however, this association lost significance after Bonferroni correction.

Factors associated with drug withdrawal are shown in Fig. 2. In multivariable analysis, drugs granted Breakthrough Therapy designation (OR 0.26; 95% CI, 0.10–0.75; p = 0.01) had higher odds of confirming their clinical benefit. Additionally, indications for genome-targeted therapies (OR 0.26; 95% CI, 0.08–0.80; p = 0.02) were more likely to be converted to regular approval.

By contrast, at the time of accelerated approval, indications supported by pivotal trials showing low benefit according to ESMO-MCBS were more likely to be withdrawn (OR, 4.63; 95% CI, 1.50–14.33; p = 0.008) compared to indications and trials without these characteristics.

Additionally, indications for which confirmatory trials had already commenced at the time of accelerated approval were associated with higher odds of confirming their benefit (OR, 0.21; 95% CI, 0.09–0.81; p = 0.02). However, after applying the Bonferroni correction for multiple comparisons, this association lost statistical significance (OR, 0.28; 95% CI, 0.64–1.19; p = 0.08).

Repeating our analysis with multivariable logistic regression, limited to variables significant in the univariate analysis, produced similar results (Supplementary Table S4).

### Temporal differences in associations

To further explore advancements in cancer treatment over the past decade, we conducted a sensitivity analysis stratified into two time periods: the most recent decade (2013–2022), which included 72 indications (25 withdrawals, 35%), and the preceding two decades (1992–2012), encompassing 54 indications (6 withdrawals, 11%).

In the multivariable analysis for the 2013–2022 period and the 1992–2017 period, and after applying the Bonferroni correction for multiple comparisons, the results were consistent with those of the overall cohort (Table 2). Notably, for the 2013–2022 period, higher withdrawal rates were significantly associated with low ESMO-MCBS scores (OR 6.66; 95% CI, 1.33–33.31; p = 0.021). Lower withdrawal rates correlated with Breakthrough Therapy designations (OR 0.25; 95% CI, 0.09–0.70; p = 0.009) and approval of genomic-targeted therapies (OR 0.20; 95% CI, 0.05–0.75; p = 0.017). Similar results were found when excluding the last 5 years (1992–2017 period), except for indications granted Breakthrough Therapy designations, which lost statistical significance. In the multivariable analyses for the 1992–2012 period, none of the variables examined were significant predictors of withdrawal.

**Table 2:** Characteristics associated with withdrawn indications by decades (2013–2022 and 1992–2012) in the multivariable logistic regression models.^a^

Variable	2013–2022		1992–2017		1992–2012	
	OR (95% CI)	p^b^	OR (95% CI)	p^b^	OR (95% CI)	p^b^
Initial approval (vs. Supplemental)	2.06 (0.58–7.33)	0.27	1.81 (0.35–9.45)	0.48	2.48 (0.48–12.59)	0.27
Orphan Drug Designation (vs. not)	1.87 (0.50–7.01)	0.36	1.57 (0.27–9.04)	0.61	2.15 (0.43–10.82)	0.35
Priority Review Designation (vs. not)	0.32 (0.03–4.05)	0.38	0.45 (0.06–3.18)	0.42	0.96 (0.18–5.02)	0.96
Breakthrough Therapy Designation (vs. not)^c^	0.25 (0.09–0.70)	0.009	0.31 (0.06–1.57)	0.16	–	–
Confirmatory study underway at the time of accelerated approval^d^	0.28 (0.06–1.19)	0.084	0.56 (0.08–4.12)	0.57	0.28 (0.06–1.43)	0.13
Hematologic (vs. solid)	1.76 (0.45–6.91)	0.42	3.45 (0.98–12.18)	0.055	3.80 (0.67–21.47)	0.13
Genome-targeted therapies (vs. non-genome targeted therapies)	0.20 (0.05–0.75)	0.017	0.10 (0.01–0.77)	0.027	0.29 (0.31–2.68)	0.27
Immune checkpoint Inhibitor (vs. non-immune checkpoint inhibitor)^e^	0.69 (0.17–2.81)	0.61	2.60 (0.60–11.38)	0.020	–	–
One trial supporting accelerated approval (vs. multiple trials)	0.37 (0.41–3.36)	0.38	0.60 (0.17–2.11)	0.42	0.58 (0.08–4.32)	0.60
Number of patients	1.01 (0.99–1.02)	0.12	1.01 (0.99–1.01)	0.24	1.00 (0.99–1.07)	0.45
Randomized controlled trial (vs. single-arm trial)	0.68 (0.18–2.51)	0.56	0.77 (0.19–3.14)	0.71	0.93 (0.07–1.09)	0.90
Phase I-II (vs. phase III)^f^	2.08 (0.19-23.31)	0.55	3.22 (0.47–22.11)	0.24	–	–
Time to event endpoint (vs. not)	0.90 (0.18–4.67)-	0.90	2.27 (0.35–14.70)	0.39	0.28 (0.01-7.69)	0.45
ESMO-MCBS low clinical benefit (vs. intermediate and high benefit)^g^	6.66 (1.33–33.31)	0.021	14.4 (1.48–140.97)	0.022	–	–

a Based on multivariable logistic regression. p values are two-sided. Significance was initially set at p < 0.05 and adjusted using Bonferroni correction for multiple comparisons.

b This analysis included 79 accelerated approvals from 2013 to 2022 and 54 accelerated approvals from 1992 to 2012.

c Multivariable analysis was not feasible due to the limited number of variables available from 1992 to 2012. Introduced by the FDA in July 2012, no drug approved via the accelerated approval pathway before December 2012 received the Breakthrough Therapy designation.

d Indications with confirmatory trials underway at the time of accelerated approval were initially associated with lower odds of withdrawal (OR 0.21; 95% CI, 0.06–0.81; p = 0.02), but this lost significance after Bonferroni correction.

e Multivariable analysis was not feasible due to the limited number of variables available from 1992 to 2012. The first immune checkpoint inhibitor via accelerated approval was approved on September 4, 2014, for refractory, unresectable, or metastatic melanoma.

f Multivariable analysis was infeasible due to limited variables from 1992 to 2012, with only one of six pivotal trials for withdrawn indications being phase III.

g Multivariable analysis was not feasible due to the limited number of variables available from 1992 to 2012. During this period, none of the 6 pivotal trials supporting the withdrawn indications met the ESMO-MCBS criteria for intermediate or high benefit (4 with low benefit and 2 unscorable).

## Discussion

This study identifies key factors associated with the withdrawal of indications approved through the accelerated approval pathway over the past three decades. Our findings indicate that drugs approved based on pivotal trials with low clinical benefit, as measured by validated scales such as the ESMO-MCBS, were more prone to market withdrawal. Factors like Breakthrough Therapy designation and indications supporting genomic-targeted therapies were linked to a reduced risk of withdrawal. In the most recent decade, these factors effectively identified therapies with genuine clinical benefit, underscoring the evolution in factors influencing drug withdrawal in oncology. These insights can aid regulators in refining the selection process for therapies undergoing accelerated approval and provide valuable guidance to patients and physicians when considering the use of such treatments.

In March 2023, the FDA introduced new draft guidance^28^ to optimize clinical trial design for oncology therapies seeking accelerated approval. This guidance recommends that, in addition to statistical significance, pivotal trials should also evaluate “clinical meaningfulness,” although this concept is not defined further. Our findings suggest that drugs demonstrating low initial benefit, based on the ESMO-MCBS scale at the time of accelerated approval, are more prone to withdrawal. This highlights the potential of value frameworks in identifying drugs with low or uncertain clinical benefit, helping to improve patient and regulatory decision-making relating to these drugs. In response to the rising costs of cancer drugs and the disconnect between their price, affordability, and clinical value,^29^ ESMO has developed and continuously updates its ESMO-MCBS tool.^24^ This scale evaluates a drug's clinical impact by considering factors such as median survival differences between treatment groups, hazard ratios, toxicity profiles, and quality of life effects. Unlike other scales,^30^^,^^31^ it also enables the evaluation of single-arm studies.

One of the key aspects of the new draft guidance is its focus on enhancing data quality and improving clinical trial efficiency by prioritizing randomized controlled trials over single-arm trials, which have frequently been used to support accelerated approvals.^32^ Our study shows that nearly 70% of accelerated approvals have been based on single-arm studies assessing preliminary efficacy in small, specific patient cohorts. Among randomized trials, only 23% were double-blind, and most evaluated changes to surrogate measures. Just 2 pivotal trials (1%) demonstrated an overall survival benefit, leading to accelerated approval. The limited methodological rigor observed in pivotal trials supporting accelerated approvals—such as the lack of randomization and small sample sizes—underscores the need for more robust trial designs to ensure reliable outcomes when confirming clinical benefit. These findings also highlight the gap between past approval practices and the challenges involved in implementing the new guidance, which recommends that sponsors initiate a randomized trial in an earlier line of treatment. Thus, accelerated approval would be based on intermediate overall response rates, while traditional approval would require demonstrating clinical benefit, such as improved overall survival, through extended follow-up within the same study. An alternative option would be to conduct two simultaneous studies: a single-arm study for heavily pretreated patients, serving as the basis for accelerated approval, and a concurrent randomized trial for patients at an earlier stage. If both studies are conducted simultaneously, interim safety and response data from the confirmatory trial could support accelerated approval, and if the overall response rates are significant, additional indications could also be approved. This approach would provide a more robust safety assessment through randomization, mitigate the risk of premature drug discontinuation, and minimize concerns about clinical equipoise, ascertainment bias, inadequate recruitment, and concerns over standard-care or placebo assignments in confirmatory trials following accelerated approval.

Since its inception, 19% of oncology indications granted under the accelerated approval pathway have been withdrawn, primarily due to the absence of verified clinical benefit, unresolved safety or efficacy concerns, and/or insufficient confirmatory clinical data. In most cases, companies voluntarily withdraw an accelerated approval indication after discussions with the FDA or in response to an unfavorable advisory committee review. However, only two withdrawals have necessitated formal hearings or appeals: the 2011 withdrawal of bevacizumab for metastatic breast cancer and the more recent withdrawal of melphalan flufenamide.^33^ Drugs may also be withdrawn due to lower-than-expected actual benefit, failure in less pretreated or broadened populations, underperformance compared to standard treatments, or high toxicity rates that compromise the benefit-risk ratio.^34^ For example, romidepsin and ibrutinib had specific indications withdrawn after confirmatory trials failed to demonstrate benefit - romidepsin seems to offer benefit only in a subtype of T-cell lymphomas that its confirmatory trial was not specificallly targeted at, and ibrutinib was withdrawn largely due to the trial design of its confirmatory trial.^35^ Similarly, immune checkpoint inhibitors approved as monotherapy frequently did not demonstrate superiority over standard chemotherapy in randomized trials, resulting in the withdrawal of 8 out of 31 accelerated approval indications due to early deaths not outweighed by observed benefits.^36^ Substantial toxicity concerns at the time of accelerated approval can lead to overestimation of treatment benefits (for example because response rate does not capture toxicity), making these drug indications more susceptible to withdrawal, as seen with the PI3K inhibitors idelalisib and duvelisib.^37^ Despite the distinct characteristics of each drug and indication, they all fail to meet the ESMO-MCBS thresholds for intermediate or high benefit when evaluating the underlying evidence.

Our study revealed that genome-targeted agents were less likely to be withdrawn. The uncertain outcomes for immune checkpoint inhibitor monotherapy contrast with the successes observed in precision oncology, where treatments target more selective populations and achieve higher objective response rates. Recent studies indicate that single-arm trials supporting accelerated approvals in precision oncology achieve a median ORR of 53%.^20^ Furthermore, earlier research shows that targeted molecular agents in randomized clinical trials not only have fewer grade 3-4 adverse events compared to controls^38^ but also demonstrate greater efficacy.^39^ Collectively, these findings may explain why indications for genome-targeted therapies were more likely to be converted to regular approval.

In addition, drugs receiving the Breakthrough Therapy designation were more likely to confirm their benefits and achieve regular approval. In 2012, the FDA established this designation to expedite the development and approval of promising treatments for serious or life-threatening conditions. A drug may qualify if preliminary clinical evidence indicates significant improvement in clinically meaningful endpoints, such as survival or surrogate markers predicting clinical benefits. This designation provides sponsors with intensive FDA guidance, facilitating quicker development and regulatory review. Although prior studies have mixed findings on efficacy superiority,^40^^,^^41^ the close FDA oversight associated with this designation may improve the likelihood of success of these drugs.

Since more than one in five drugs approved through the accelerated approval pathway for cancer indications has subsequently been withdrawn from the US market, it is imperative to refine this pathway to more accurately assess efficacy and enhance patient safety. The FDA should establish clear and precise criteria for when accelerated approval is a reasonable choice, in collaboration with sponsors and patients. For treatments that demonstrate low clinical benefit according to validated scales—due to modest response rates or safety concerns—accelerated approval based on single-arm trials may not be appropriate, particularly when alternative supportive and palliative therapies are available. In these cases, the FDA may reasonably seek additional safety data, dosage information, and results from randomized studies before approving the drugs.

Moreover, drugs with a well-understood mechanism of action and a clearly identified subgroup of patients who may benefit—such as genome-targeted cancer therapies—should be prioritized for accelerated approval. Similarly, those where preliminary clinical evidence indicates substantial improvements over existing therapies, particularly under the guidance of rigorous FDA oversight (such as drugs designated as ‘breakthrough’ therapies), potentially represent ideal candidates for this expedited pathway. By implementing these refinements, the FDA can enhance the integrity of the accelerated approval process, ultimately ensuring that patients receive treatments that are not only innovative but also safe and effective.

These findings have implications beyond the US regulatory system.^42^ International collaborations, such as the FDA-led Project Orbis, have facilitated reducing the lag in drug approvals across multiple countries, including Australia, Brazil, Canada, Israel, the UK, Singapore and Switzerland.^43^ While Project Orbis aims to expedite access to promising therapies, further evaluation is needed to understand differences in regulatory frameworks, post-market surveillance rigor, and health system priorities across participating countries, particularly regarding their influence on local withdrawal decisions.^44^

The accelerated approval program allows faster access to new therapies in the US.^45^^,^^46^ However, the program also carries important risks. Several analyses have highlighted the global repercussions of accelerated approval, especially in low- and middle-income countries that lack robust regulatory authorities or sufficient resources to independently evaluate new drugs, relying instead on FDA approval status.^47^ This reliance has led to instances when drugs without confirmed clinical benefits remain active in other jurisdictions, perpetuating the use of ineffective treatments.^7^^,^^10^

The economic and ethical implications of this issue are profound. The high costs of these drugs can render them unaffordable for many patients, potentially depriving them of effective treatment options. Furthermore, the opportunity costs of prioritizing these therapies may limit access to more suitable and efficacious alternatives, underscoring the need for stricter evaluation processes and resource allocation.^47^

This study's limitations include that not all label updates are available on the FDA website. Second, we did not consider ratings from other frameworks. Although frameworks such as the American Society of Clinical Oncology Value Framework (ASCO-VF), the American Society of Clinical Oncology Cancer Research Committee (ASCO-CRC), and the National Comprehensive Cancer Network (NCCN) Evidence Blocks exist, they are not applicable in this context. ASCO-VF and ASCO-CRC exclude evidence from single-arm trials, relying exclusively on randomized controlled trials, which limits their relevance since nearly 70% of trials supporting accelerated approvals are single-arm studies. Introduced in 2016, the NCCN Evidence Blocks rank treatments based on expert opinion, evaluating the cumulative evidence available at the time of guideline publication rather than focusing solely on pivotal trials that led to accelerated approval. Furthermore, since most drugs approved through accelerated approval (56%, 74/133) received approval before 2016, they were not evaluated under this framework. Third, the ESMO-Magnitude of Clinical Benefit Scale (ESMO-MCBS) for hematological malignancies was recently published,^25^ and scores for most treatments and diseases are not yet publicly available. For these malignancies, we relied on the ESMO-MCBS-H appendix, and when unavailable, scoring was performed independently by two authors. Fourth, to minimize missing data, we reviewed all accessible package inserts online and examined FDA approval letters and published drug registration trials cited in these inserts. Fifth, there is a high degree of heterogeneity among cancer types. The use of clinical benefit scales, which have different forms depending on whether they are applied to solid or liquid tumors, as well as in different scenarios (curative vs. non-curative) and study designs (randomized vs. single-arm), partially addresses this limitation. In this analysis, we focused solely on comparing withdrawn to converted indications; therefore, the role of accelerated indications that are still ongoing remains unexamined.

The FDA's accelerated approval program allows drug approval for serious conditions using unvalidated surrogate measures, which can lead to uncertainty about actual clinical benefits and possible failures in confirmatory trials. To address this uncertainty and enhance the likelihood that accelerated approvals will support medications optimally useful in the care of patients, regulatory bodies must implement measures that address both the uncertainty and the time frame of the transition to regular approval. Such measures are pivotal in protecting patient decision making and enhancing the practice of evidence-based medicine.

## Contributors

Drs Tibau and Romano had full access to all the data in the study and take responsibility for the integrity of the data and the accuracy of the data analysis. Study concept and design: Tibau, Kesselheim. Acquisition, analysis, or interpretation of data: Tibau, Romano, Borrell, Molto, Kesselheim. Drafting of the manuscript: Tibau. Critical revision of the manuscript for important intellectual content: Tibau, Cliff, Romano, Borrell, Molto, Kesselheim. Statistical analysis: Tibau. Administrative, technical, or material support: Tibau. Study supervision: Tibau, Cliff, Kesselheim. All authors had access to all the data, participated in revising the manuscript, and approved its final version.

## Data sharing statement

The datasets generated and analyzed during this study will be available from the corresponding author upon reasonable request after publication.

## Declaration of interests

A. Tibau: Support for attending meetings and/or travel: Daiichi Sankyo, Novartis. M. Borrell: Consulting fees: Pizer; Payment of honoraria for lectures, presentations, educational events: Pizer, Lilly, Gilead; Support for attending meetings and/or travel: Pizer, Lilly, Gilead, Novartis. C. Molto: Payment of honoraria for lectures, presentations, educational events: AstraZeneca, Merck. All remaining authors have declared no conflicts of interest.
