# Supplementary material for: Predictors of withdrawal of anticancer drug indications granted accelerated approval: a retrospective cohort study
Source: eClinicalMedicine. 2025 May 31;84:103088. doi: 10.1016/j.eclinm.2025.103088 (PMC12273735; doi:10.1016/j.eclinm.2025.103088)
Supplement: Supplementary Tables [file mmc1.docx]

**Supplementary material for:**

Identifying Predictors of Withdrawal of Anticancer Drug Indications Granted Accelerated Approval

Ariadna Tibau, MD, PhD^1,2,3,4^, Edward R Scheffer Cliff, MD^1,2,5^, Alejandra Romano, MD^3, 4^, Maria Borrell, MD^6^, Consolacion Molto, MD, PhD ^7, 8, 9^, Aaron S Kesselheim, MD, JD, MPH^1, 2^

Affiliations: ^1^ Program On Regulation, Therapeutics, And Law (PORTAL), Division of Pharmacoepidemiology and Pharmacoeconomics, Department of Medicine, Brigham and Women's Hospital, Boston, Massachusetts, USA. ^2^ Harvard Medical School, Boston, Massachusetts, USA. ^3^Oncology Department, Hospital de la Santa Creu i Sant Pau, Institut d’Investigació Biomèdica Sant Pau.

^4^ Departament of Medicine, Universitat Autònoma de Barcelona, Barcelona, Catalonia, Spain. ^5^Department of Clinical Haematology, Peter MacCallum Cancer Centre and Royal Melbourne Hospital, Melbourne, Australia.^6^ Vall d'Hebron Institute of Oncology (VHIO), Barcelona, Spain; Medical Oncology Department, Vall d'Hebron Hospital, Barcelona, Spain. ^7^ R.S. McLaughlin Durham Regional Cancer Centre, Oshawa, Ontario, Canada. ^8^ Department of Oncology, Queen's University, Kingston, ON, Canada. ^9^ Division of Cancer Care and Epidemiology, Queen's Cancer Research Institute, Kingston, ON, Canada.

**Index**

**Supplementary Methods**

STROBE Checklist for Reporting Observational Studies

**Supplementary Results**

**Supplementary Table 1.**  ESMO-MCBS version 1.1 and ESMO-MCBS:H version 1.0 levels of evidence for solid and hematologic cancers in curative and non-curative settings

**Supplementary Table 2.** Accelerated Approvals for Malignant Hematology and Oncology Products That Verified Benefit

**Supplementary Table 3.** Accelerated Approvals for Malignant Hematology and Oncology Products That Were Withdrawn

**Supplementary Table 4.** Characteristics associated with Withdrawn Indications in the Univariable and Sensitivity Multivariable Logistic Regression Model.

STROBE Checklist for Reporting Observational Studies

|  | Item No | Recommendation | Assessment and Methods for Variables of Interest |
| --- | --- | --- | --- |
| **Title and abstract** | 1 | (*a*) Indicate the study’s design with a commonly used term in the title or the abstract | The study design is indicated as "retrospective cohort study" in the abstract. |
|  |  | (*b*) Provide in the abstract an informative and balanced summary of what was done and what was found | The abstract summarizes the study’s purpose (identifying factors associated with drug withdrawal after accelerated approval) and key findings (Breakthrough Therapy designation and genome-targeted therapies linked to lower withdrawal rates, while low ESMO-MCBS scores linked to higher rates). |
| Introduction | | |  |
| Background/rationale | 2 | Explain the scientific background and rationale for the investigation being reported | The introduction highlights the FDA's accelerated approval pathway, emphasizing its role in providing timely access to cancer treatments. It discusses the use of surrogate measures, the need for confirmatory studies, and concerns about delayed trials and slow withdrawals, setting the stage for the investigation of these challenges. |
| Objectives | 3 | State specific objectives, including any prespecified hypotheses | The study aims to identify factors linked to the withdrawal of oncology drug indications after accelerated approval, focusing on regulatory pathways, trial characteristics, clinical benefit assessments, and confirmatory study initiation. These objectives address key predictors of withdrawal. |
| Methods | | |  |
| Study design | 4 | Present key elements of study design early in the paper | This study is a cohort study analyzing cancer drugs granted accelerated approval by the FDA from 1992 to 2022. It aims to identify factors influencing the withdrawal of oncology drug indications after accelerated approval. |
| Setting | 5 | Describe the setting, locations, and relevant dates, including periods of recruitment, exposure, follow-up, and data collection | Setting and Locations: The study focuses on cancer drugs approved under the FDA's accelerated approval pathway in the U.S.  Relevant Dates - Periods of Recruitment, Exposure, and Follow-up:  -Recruitment: Oncology drugs granted accelerated approval between 1992 and 2022.  -Exposure: Drugs approved under accelerated approval, with follow-up on confirmatory trials and withdrawals.  -Follow-up: Monitoring confirmatory trials and drug status (withdrawn or fully approved) until July 15, 2024. |
| Participants | 6 | (*a*) *Cohort study*—Give the eligibility criteria, and the sources and methods of selection of participants. Describe methods of follow-up  *Case-control study*—Give the eligibility criteria, and the sources and methods of case ascertainment and control selection. Give the rationale for the choice of cases and controls  *Cross-sectional study*—Give the eligibility criteria, and the sources and methods of selection of participants | This aligns with the cohort study design, which tracks exposure (accelerated approval) and its outcomes (confirmatory trials and drug status) over time. |
|  |  | (*b*) *Cohort study*—For matched studies, give matching criteria and number of exposed and unexposed  *Case-control study*—For matched studies, give matching criteria and the number of controls per case | In this cohort study, matching was not explicitly applied. Instead, the analysis focused on comparing drug indications with different characteristics, such as approval pathways, therapeutic types, and clinical trial characteristics. |
| Variables | 7 | Clearly define all outcomes, exposures, predictors, potential confounders, and effect modifiers. Give diagnostic criteria, if applicable | Outcomes:  -Drug withdrawal: Whether a cancer drug granted accelerated approval is withdrawn from the market.  -Conversion to full approval: Transition from accelerated approval to regular approval.  -Clinical benefit: Assessed using the ESMO-MCBS scale (substantial, intermediate, low).  Exposures:  -Accelerated approval: Approval via specific FDA pathways (Priority Review, Breakthrough Therapy, Orphan Drug).  -Confirmatory studies: Status at the time of accelerated approval (initiated, delayed, or completed).  Predictors:  -Regulatory pathways: Different FDA approval pathways.  -Therapeutic drug type: Cytotoxic, immune checkpoint inhibitors, targeted therapies.  -Clinical trial characteristics: Trial number, sample size, design, and phase.  -ESMO-MCBS score: Clinical benefit score.  Potential Confounders:  -Approval period: Variations in FDA practices over time.  -Therapeutic class: Differences in drug types and their approval outcomes.  -Trial sample size: Larger trials may affect outcomes.  Effect Modifiers:  -Drug type: Different classes of drugs may have distinct withdrawal rates.  -Confirmatory study status: The completion and timing of confirmatory trials may modify outcomes.  Diagnostic Criteria:  -ESMO-MCBS: Grades A/B (substantial), 3 (intermediate), 1/2/C (low).  -Withdrawal: Withdrawal of drugs after accelerated approval.  -Confirmatory study status: Verifying trial initiation and completion. |
| Data sources/ measurement | 8* | For each variable of interest, give sources of data and details of methods of assessment (measurement). Describe comparability of assessment methods if there is more than one group | Drug Information (Accelerated Approval, Conversion, or Withdrawal):  -Source: FDA website  -Assessment: Identification of drugs that received accelerated approval from 1992–2022, with status checked through July 2024 for withdrawal or conversion to regular approval.  -Comparability: Data were reviewed by at least two authors to ensure consistency and accuracy across groups.  Clinical Trial Characteristics (Number, Sample Size, Design, Phase, Endpoints):  -Source: FDA drug labels and clinical trial reports  -Assessment: Extraction of data on pivotal trials, trial phases (1-2 vs. 3), designs (randomized vs. single-arm), and primary endpoints (overall survival vs. intermediate/non-time-to-event).  -Comparability: Trials supporting accelerated approvals were prioritized by endpoint strength, ensuring comparability between trials with similar endpoints.  Clinical Benefit (ESMO-MCBS Scale):  -Source: ESMO-MCBS scale (version 1.1 for solid cancers, version 1.0 for hematological cancers)  -Assessment: Scoring based on trial outcomes (A/B for substantial, 3 for intermediate, and 1/2/C for low).  -Comparability: Standardized assessment through ESMO-MCBS for both solid and hematological cancers, ensuring consistency across cancer types.  Therapeutic Drug Type (Cytotoxic, Immune Checkpoint Inhibitors, Targeted Therapies):  -Source: FDA labels and drug characteristics  -Assessment: Categorization based on drug type and mechanism of action.  -Comparability: Grouping by drug type ensures comparability within therapeutic categories across groups.  Confirmatory Studies Status:  -Source: ClinicalTrials.gov  -Assessment: Verified the status of confirmatory studies for drugs at the time of accelerated approval.  -Comparability: Data on confirmatory studies were consistent across drugs from the same regulatory pathways. |
| Bias | 9 | Describe any efforts to address potential sources of bias | Efforts to address potential sources of bias included data review by at least two authors and cross-referencing data from the FDA and ClinicalTrials.gov to ensure accuracy. |
| Study size | 10 | Explain how the study size was arrived at | The study size was determined by including all oncology drugs approved under the FDA's accelerated approval program between 1992 and 2022, focusing on those with available confirmatory trial data and clinical outcomes. |
| Quantitative variables | 11 | Explain how quantitative variables were handled in the analyses. If applicable, describe which groupings were chosen and why | Quantitative variables, like pivotal trials, sample sizes, and clinical benefit grades, were summarized using proportions, medians, and interquartile ranges. Clinical benefit was grouped by the ESMO-MCBS scale: substantial (grades A/B for curative, 4/5 for palliative), intermediate (grade 3), and low (grades 1/2/C), prioritizing survival and robust endpoints. |
| Statistical methods | 12 | (*a*) Describe all statistical methods, including those used to control for confounding | Logistic regression identified factors linked to drug withdrawal. Univariate and multivariable analyses were conducted. Models controlled for confounding by selecting one variable per 10 events to prevent overfitting. |
|  |  | (*b*) Describe any methods used to examine subgroups and interactions | A sensitivity analysis compared data from 2013–2022 to 1992–2012 to assess trends in new cancer therapies. |
|  |  | (*c*) Explain how missing data were addressed | The handling of missing data was not specified, as the cases were complete for nearly all analyses. |
|  |  | (*d*) *Cohort study*—If applicable, explain how loss to follow-up was addressed  *Case-control study*—If applicable, explain how matching of cases and controls was addressed  *Cross-sectional study*—If applicable, describe analytical methods taking account of sampling strategy | Loss to follow-up was not an issue, as the study used publicly available data. |
|  |  | (*e*) Describe any sensitivity analyses | Sensitivity analysis repeated regression with significant univariate variables. An additional analysis compared 2013–2022 with 1992–2012 to assess therapy impacts. |

Continued on next page

| Results | | |
| --- | --- | --- |
| Participants | 13* | (a) Report numbers of individuals at each stage of study—eg numbers potentially eligible, examined for eligibility, confirmed eligible, included in the study, completing follow-up, and analysed |
|  |  | (b) Give reasons for non-participation at each stage |
|  |  | (c) Consider use of a flow diagram |
| Descriptive data | 14* | (a) Give characteristics of study participants (eg demographic, clinical, social) and information on exposures and potential confounders |
|  |  | (b) Indicate number of participants with missing data for each variable of interest |
|  |  | (c) *Cohort study*—Summarise follow-up time (eg, average and total amount) |
| Outcome data | 15* | *Cohort study*—Report numbers of outcome events or summary measures over time |
|  |  | *Case-control study—*Report numbers in each exposure category, or summary measures of exposure |
|  |  | *Cross-sectional study—*Report numbers of outcome events or summary measures |
| Main results | 16 | (*a*) Give unadjusted estimates and, if applicable, confounder-adjusted estimates and their precision (eg, 95% confidence interval). Make clear which confounders were adjusted for and why they were included |
|  |  | (*b*) Report category boundaries when continuous variables were categorized |
|  |  | (*c*) If relevant, consider translating estimates of relative risk into absolute risk for a meaningful time period |
| Other analyses | 17 | Report other analyses done—eg analyses of subgroups and interactions, and sensitivity analyses |
| Discussion | | |
| Key results | 18 | Summarise key results with reference to study objectives |
| Limitations | 19 | Discuss limitations of the study, taking into account sources of potential bias or imprecision. Discuss both direction and magnitude of any potential bias |
| Interpretation | 20 | Give a cautious overall interpretation of results considering objectives, limitations, multiplicity of analyses, results from similar studies, and other relevant evidence |
| Generalisability | 21 | Discuss the generalisability (external validity) of the study results |
| Other information | | |
| Funding | 22 | Give the source of funding and the role of the funders for the present study and, if applicable, for the original study on which the present article is based |

*Give information separately for cases and controls in case-control studies and, if applicable, for exposed and unexposed groups in cohort and cross-sectional studies.

**Note:** An Explanation and Elaboration article discusses each checklist item and gives methodological background and published examples of transparent reporting. The STROBE checklist is best used in conjunction with this article (freely available on the Web sites of PLoS Medicine at http://www.plosmedicine.org/, Annals of Internal Medicine at http://www.annals.org/, and Epidemiology at http://www.epidem.com/). Information on the STROBE Initiative is available at www.strobe-statement.org.

**Supplementary Table 1.**

ESMO-MCBS version 1.1^1^ and ESMO-MCBS:H version 1.0^2^ levels of evidence for solid and hematologic cancers in curative and non-curative settings

| **Calculate Aggregate Value (Formulation)** | **Form 1 (ESMO-MCBS)**  **Form 1 (1a, 1b ESMO-MCBS:H)** | **Form 2 (2a, 2b, 2c)** | **Form 3** |
| --- | --- | --- | --- |
| **Scenarios evaluated** | Treatments with curative intent | Treatments with non-curative intent | Treatments with non-curative intent in “orphan diseases” and in diseases with “high unmet need” |
| **Sources of evidence** | RCT  SAT (ESMO-MCBS:H) | RCT | SAT |
| **First step.** Preliminary score described with the evaluated outcome (primary endpoint) | DFS or OS | OS (Form 2a) PFS (Form 2b) QoL, ORR, AE (Form 2c) | PFS or ORR |
| **Second step.** Positive (score upgrade) and negative (score downgrade) adjustments of the preliminary score | 1. Acut transient and persistent toxicity (Form 1a and 1b). | 1. QoL improved or worsened.  2. AE that impact daily well-being.  3. Incremental toxicities that result in hospitalization (Form 2b).  4. Plateau (Form 2a, 2b)  5. OS and PFS benefit (Form 2b)  6. Early crossover (Form 2b). | 1. QoL improved or worsened.  2. Side effects that impact daily well being.  3. Phase 4 experience |
| **Grading** | A, B, C | 1,2,3,4,5 | 1,2,3,4 |
| **Substantial magnitude of clinical benefit** | A, B | 4,5* | 4 |
| **Examples:** | *ACOSOG Z9001,^3^ GIST KIT+*  Preliminary-score:  RCT RFS  RFS Control: 83%  RFS Gain: 15%  RFS HR 0.35 (0.22-0.53)  QoL adjustment: Not qualified for an ESMO-MCBS benefit.  Final score: A. | *E2100,^4^ HER2- BC*  Preliminary-score:  RCT PFS (Form 2b):  PFS Control: 5.9 months  PFS Gain: 5.9 months  PFS HR 0.60 (0.51-0.70)  QoL adjustment: No QoL benefit.  Preliminary score: 3.  Final score: 2 (Improved PFS without QoL or OS benefit) | *DESTINY-Breast01,^5^ HER2+, BC*  Preliminary-score:  Non-curative SAT ORR (Form 3):  ORR: 60.9%  DOR: 14.8 months  Adjustments. Toxicity: 52.2% Grade 3  2% Grade 5.  Preliminary score: 3.  Final score: 2. |

Abbreviations: AE, adverse events; BC, breast cancer; DFS, Disease-Free Survival; DOR, duration of response; ESMO-MCBS, European Society for Medical Oncology-Magnitude of Clinical Benefit Scale; ESMO-MCBS:H, ESMO-MCBS for Haematological Malignancies; GIST, GastroIntestinal Stromal Tumor; HER2, Human Epidermal growth factor Receptor 2; OS, overall survival; PFS, progression-free survival, PROs, Patient Reported Outcomes; QoL, Quality of life, RCT, randomized controlled trials, SAT, Single-arm trials; TTD, Time to deterioration.

* A score of 5 is only achievable for therapies with a primary endpoint of OS (Form 2a)

**References**

1 Cherny NI, Dafni U, Bogaerts J, *et al.* ESMO-Magnitude of Clinical Benefit Scale version 1.1. *Annals of Oncology* 2017; 28: 2340–66.

2 Kiesewetter B, Dafni U, de Vries EGE, *et al.* ESMO-Magnitude of Clinical Benefit Scale for haematological malignancies (ESMO-MCBS:H) version 1.0. *Annals of Oncology* 2023; 34: 734–71.

3 Dematteo RP, Ballman KV, Antonescu CR, *et al.* Adjuvant imatinib mesylate after resection of localised, primary gastrointestinal stromal tumour: a randomised, double-blind, placebo-controlled trial. *The Lancet* 2009; 1097-1104.

4 Miller K, Wang, M, Gralow J, *et al.* Paclitaxel plus Bevacizumab versus Paclitaxel Alone for Metastatic Breast Cancer. *N Engl J Med* 2007;357:2666-2676.

5 Modi S, Saura C, Yamashita T, *et al.* Trastuzumab Deruxtecan in Previously Treated HER2-Positive Breast Cancer. *New England Journal of Medicine* 2020; 382: 610–21.

|  | | | |
| --- | --- | --- | --- |
| **Supplementary Table 2.** Accelerated Approvals for Malignant Hematology and Oncology Products That Verified Benefit | | | |
| **Product** | **Original Accelerated Approval Indication** | **Date of AA** | **Date of RA** |
| Bicalutamide (Casodex) | Advanced prostate cancer | 10/4/1995 | 12/12/1997 |
| Liposomal doxorubicin (Doxil) | Refractory AIDS-related Kaposi's sarcoma | 11/17/1995 | 6/10/2008 |
| Docetaxel (Taxotere) | Refractory locally advanced or metastatic BC | 5/14/1996 | 6/22/1998 |
| Irinotecan hcl trihydrote (Camptosar) | Refractory metastatic CRC | 6/14/1996 | 10/22/1998 |
| Capecitabine (Xeloda) | Refractory Metastatic BC | 4/30/1998 | 9/7/2001 |
| Denileukin diftitox (Ontak) | CD25+ refractory CTCL | 2/5/1999 | 10/15/2008 |
| Cytarabine liposomal (Depocyt) | Lymphomatous meningitis | 4/1/1999 | 4/19/2007 |
| Liposomal doxorubicin (Doxil) | Refractory metastatic ovarian carcinoma | 6/28/1999 | 1/28/2005 |
| Temozolomide Temodar | Adults with refractory anaplastic astrocytoma | 8/11/1999 | 3/15/2005 |
| Alemtuzumab (Campath) | Refractory B-cell CLL | 5/7/2001 | 9/19/2007 |
| Imatinib mesylate (Gleevec) | CML in blast crisis, AP, or in CP after failure of interferon-alpha | 5/10/2001 | 12/8/2003 |
| Imatinib mesylate (Gleevec) | KIT (CD117) positive unresectable and/or metastatic GIST | 2/1/2002 | 9/26/2008 |
| Ibritumomab tiuxetan (Zevalin) | Refractory low-grade follicular or transformed B-cell NHL | 2/19/2002 | 9/3/2009 |
| Oxaliplatin (Eloxatin) | Refractory metastatic CRC | 8/9/2002 | 1/9/2004 |
| Anastrozole (Arimidex) | Adjuvant, postmenopausal, HR+ early BC | 9/5/2002 | 9/16/2005 |
| Imatinib mesylate (Gleevec) | Newly diagnosed adults with Ph+ CML | 12/20/2002 | 5/27/2009 |
| Bortezomib (Velcade) | Refractory MM | 5/13/2003 | 3/25/2005 |
| Imatinib mesylate (Gleevec) | Pediatric patients, refractory PH+ CP CML | 5/20/2003 | 9/27/2006 |
| Cetuximab (Erbitux) | As a single agent, EGFR+ refractory metastatic CRC | 2/12/2004 | 10/2/2007 |
| Cetuximab (Erbitux) | EGFR+ refractory metastatic CRC | 2/12/2004 | 7/6/2012 |
| Pemetrexed disodium (Alimta) | Refractory metastatic NSCLC | 8/19/2004 | 7/2/2009 |
| Letrozole (Femara) | Adjuvant, postmenopausal women, HR+ BC, extended therapy. | 10/29/2004 | 4/30/2010 |
| Clofarabine (Clolar) | Pediatric, refractory ALL | 12/28/2004 | 7/18/2022 |
| Nelarabine (Arranon) | Refractory T-cell ALL or T-cell lymphoblastic lymphoma | 10/28/2005 | 7/31/2019 |
| Letrozole (Femara) | Adjuvant, postmenopausal women, HR+ BC | 12/28/2005 | 4/30/2010 |
| Sunitinib maleate (Sutent) | Advanced RCC | 1/26/2006 | 2/2/2007 |
| Thalidomide (Thalomid) | Newly diagnosed MM | 5/25/2006 | 6/19/2014 |
| Dasatinib (Sprycel) | Refractory CML | 6/28/2006 | 5/21/2009 |
| Panitumumab (Vectibix) | Refractory EGFR+ metastatic CRC | 9/27/2006 | 5/23/2014 |
| Imatinib mesylate (Gleevec) | Newly diagnosed Ph+ CML in pediatric patients | 9/27/2006 | 4/1/2011 |
| Nilotinib (Tasigna) | Refractory CP and AP Ph+ CML | 10/29/2007 | 1/14/2011 |
| Pemetrexed disodium (Alimta) | Newly diagnosed or refractory non-squamous NSCLC | 9/26/2008 | 7/2/2009 |
| Imatinib mesylate (Gleevec) | Adjuvant KIT+ GIST | 12/19/2008 | 1/31/2012 |
| Bevacizumab (Avastin) | Refractory glioblastoma | 5/5/2009 | 12/5/2017 |
| Ofatumumab (Arzerra) | Refractory CLL | 10/26/2009 | 4/17/2014 |
| Lapatinib (Tykerb) | Post-menopausal HR+/HER2+ metastatic BC | 1/29/2010 | 12/6/2018 |
| Nilotinib (Tasigna) | Newly diagnosed, Ph+ CML in CP | 6/17/2010 | 1/27/2015 |
| Dasatinib (Sprycel) | Newly diagnosed Ph+ CML in CP | 10/28/2010 | 8/12/2015 |
| Everolimus (Afinitor) | TSC who have SEGA not candidates for curative surgical resection | 10/29/2010 | 1/29/2016 |
| Brentuximab vedotin (Adcetris) | Refractory HL | 8/19/2011 | 8/17/2015 |
| Brentuximab vedotin (Adcetris) | Refractory systemic anaplastic large cell lymphoma | 8/19/2011 | 3/20/2018 |
| Crizotinib (Xalkori) | Refractory ALK+ metastatic NSCLC | 8/26/2011 | 11/20/2013 |
| Everolimus (Afinitor) | Renal angiomyolipoma associated with TSC | 4/26/2012 | 2/18/2016 |
| Carfilzomib (Kyprolis) | Refractory MM | 7/20/2012 | 1/21/2016 |
| Omacetaxine mepesuccinate (Synribo) | Refractory CML with CP or AP | 10/26/2012 | 2/10/2014 |
| Ponatinib (Iclusig) | Refractory CML in CP, AP, or BP or Refractory Ph+ ALL | 12/14/2012 | 11/28/2016 |
| Pomalidomide (Pomalyst) | Refractory MM | 2/8/2013 | 4/23/2015 |
| Pertuzumab (Perjeta) | Neoadjuvant HER2+ BC treatment | 9/30/2013 | 12/20/2017 |
| Dabrafenib (Tafinlar) | Unresectable or metastatic melanoma with BRAF V600E/K mutations | 1/9/2014 | 11/20/2015 |
| Trametinib (Mekinist) | Unresectable or metastatic melanoma with BRAF V600E/K mutations | 1/8/2014 | 11/20/2015 |
| Ibrutinib (Imbruvica) | Refractory CLL | 2/12/2014 | 7/28/2014 |
| Ceritinib (Zykadia) | Refractory ALK+ metastatic NSCLC | 4/29/2014 | 5/26/2017 |
| Pembrolizumab (Keytruda) | Refractory unresectable or metastatic melanoma | 9/4/2014 | 12/18/2015 |
| Blinatumomab (Blincyto) | Refractory Ph- or B-cell precursor ALL | 12/3/2014 | 7/11/2017 |
| Olaparib (Lynparza) | Refractory germline BRCA-mutated advanced ovarian cancer | 12/19/2014 | 8/17/2017 |
| Nivolumab (Opdivo) | Refractory unresectable or metastatic melanoma | 12/22/2014 | 3/7/2019 |
| Palbociclib (Ibrance) | Postmenopausal HR+ newly diagnosed metastatic BC | 2/3/2015 | 3/31/2017 |
| Nivolumab (Opdivo) | BRAF V600 wild-type unresectable or metastatic melanoma | 9/30/2015 | 3/7/2019 |
| Pembrolizumab (Keytruda) | Refractory metastatic PD-L1 + NSCLC | 10/2/2015 | 10/24/2016 |
| Osimertinib (Tagrisso) | Refractory metastatic EGFR T790M mutation-positive NSCLC. | 11/13/2015 | 3/30/2017 |
| Daratumumab (Darzalex) | Refractory MM | 11/16/2015 | 11/21/2016 |
| Alectinib (Alecensa) | Refractory ALK+ metastatic NSCLC | 12/11/2015 | 11/6/2017 |
| Venetoclax (Venclexta) | Refractory CLL with 17P deletion | 4/11/2016 | 6/8/2018 |
| Pembrolizumab (Keytruda) | Refractory HNSCC | 8/5/2016 | 6/10/2019 |
| Rucaparib (Rubraca) | BRCA-mutated refractory advanced ovarian cancer | 12/19/2016 | 4/6/2018 |
| Nivolumab (Opdivo) | Refractory advanced UC | 2/2/2017 | 8/19/2021 |
| Pembrolizumab (Keytruda) | Refractory classical HL | 3/14/2017 | 10/14/2020 |
| Avelumab (Bavencio) | Metastatic MCC | 3/23/2017 | 9/6/2023 |
| Brigatinib (Alunbrig) | Refractory ALK+ metastatic NSCLC | 4/28/2017 | 5/22/2020 |
| Avelumab (Bavencio) | Refractory advanced UC | 5/9/2017 | 6/30/2020 |
| Pembrolizumab (Keytruda) | Newly diagnosed metastatic non-squamous NSCLC | 5/10/2017 | 8/20/2018 |
| Pembrolizumab (Keytruda) | Advanced UC ineligible for cisplatin-containing chemotherapy | 5/18/2017 | 8/31/2021 |
| Pembrolizumab (Keytruda) | Refractory metastatic, MSI-H or dMMR solid tumors or CRC | 5/23/2017 | 3/28/2023 |
| Bosutinib (Bosulif) | Newly diagnosed Ph+ CML in CP | 12/19/2017 | 5/14/2021 |
| Blinatumomab (Blincyto) | CD19+ B-cell precursor ALL in first/second complete remission with MRD ≥0.1% | 3/29/2018 | 6/20/2023 |
| Pemetrexed disodium (Alimta) | Newly diagnosed metastatic non-squamous NSCLC | 6/4/2018 | 1/30/2019 |
| Pembrolizumab (Keytruda) | Refractory primary mediastinal large B-cell lymphoma | 6/13/2018 | 10/14/2020 |
| Pembrolizumab (Keytruda) | Refractory PD-L1+ metastatic cervical cancer | 6/12/2018 | 10/13/2021 |
| Lorlatinib (Lorbrena) | Refractory ALK+ metastatic NSCLC | 11/2/2018 | 3/3/2021 |
| Pembrolizumab (Keytruda) | Refractory HCC | 11/9/2018 | 1/25/2024 |
| Venetoclax (Venclexta) | Newly diagnosed AML | 11/21/2018 | 10/16/2020 |
| Pembrolizumab (Keytruda) | Recurrent locally advanced or metastatic MCC | 12/19/2018 | 10/12/2023 |
| Erdafitinib (Balversa) | Refractory advanced UC, with FGFR3/FGFR2 genetic alterations | 4/12/2019 | 1/19/2024 |
| Polatuzumab  vedotin- piiq (Polivy) | Refractory DLBCL | 6/10/2019 | 4/19/2023 |
| Selinexor (Xpovio) | Refractory MM | 7/3/2019 | 12/18/2020 |
| Pembrolizumab (Keytruda) | Refractory advanced endometrial carcinoma not MSI-H or dMMR | 9/17/2019 | 7/21/2021 |
| Lenvatinib (Lenvima) | Refractory advanced endometrial carcinoma not MSI-H or dMMR | 9/17/2019 | 7/21/2021 |
| Enfortumab vedotin-ejfv (Padcev) | Refractory advanced UC | 12/18/2019 | 7/9/2021 |
| Fam-trastuzumab deruxtecan-nxki (Enhertu) | Refractory metastatic HER2+ | 12/20/2019 | 5/4/2022 |
| Sacituzumab govitecan-hziy (Trodelvy) | Refractory metastatic TNBC | 4/22/2020 | 4/7/2021 |
| Capmatinib (Tabrecta) | Metastatic NSCLC with MET exon 14 skipping alterations | 5/6/2020 | 8/10/2022 |
| Selpercatinib (Retevmo) | Metastatic RET fusion+ NSCLC | 5/8/2020 | 9/21/2022 |
| Selpercatinib (Retevmo) | Advanced RET fusion+ thyroid cancer | 5/8/2020 | 6/12/2024 |
| Pralsetinib (Gavreto) | Metastatic RET fusion+ NSCLC | 9/4/2020 | 8/9/2023 |
| Pembrolizumab (Keytruda) | Locally recurrent unresectable or metastatic PD-L1+ TNBC | 11/13/2020 | 7/26/2021 |
| Tepotinib (Tepmetko) | Metastatic NSCLC with MET exon 14 skipping alterations | 2/3/2021 | 2/15/2024 |
| Cemiplimab-rwlc (Libtayo) | Refractory metastatic BCC | 2/9/2021 | 4/28/2023 |
| Dostarlimab-gxly (Jemperli) | Refractory dMMR advanced EC | 4/22/2021 | 2/9/2023 |
| Amivantamab-vmjw (Rybrevant) | Refractory advanced NSCLC with EGFR exon 20 insertion mutations | 5/21/2021 | 3/1/2024 |
| Tisotumab vedotin-tftv (Tivdak) | Refractory metastatic cervical cancer | 9/20/2021 | 4/29/2024 |
| Asciminib (Scemblix) | Refractory Ph+ CML in CP | 10/29/2021 | 10/12/2022 |
| Mirvetuximab soravtansine-gynx (Elahere) | Refractory FRα+, ovarian, fallopian tube, or primary peritoneal cancer | 11/14/2022 | 3/22/2024 |
| Abbreviations: AA, accelerated approval; AIDS, acquired immunodeficiency syndrome; ALK, anaplastic lymphoma receptor tyrosine kinase; ALL, acute lymphocytic leukemia; AML, Acute Myeloid Leukemia; AP, accelerated phase; ASCT, autologous stem cell transplant; BCC, basal cell carcinoma; BC, breast cancer; BP, blast phase; BRAF, B-Raf proto-oncogene, serine/threonine kinase; HL, Hodgkin Lymphoma; CLL, chronic lymphocytic leukemia; CML, chronic myelogenous leukemia; CRC, colorectal cancer; CP, chronic phase; CTCL, cutaneous T-cell lymphoma; EC, endometrial cancer; EGFR, epidermal growth factor receptor; DLBCL, diffuse large B-cell lymphoma; dMMR, mismatch repair deficient; EGFR, epidermal growth factor receptor; FRα, folate receptor-alpha; GIST, gastrointestinal stromal tumor; FGFR2, fibroblast growth factor receptor 2; FGFR3, fibroblast growth factor receptor 3; HCC, hepatocellular carcinoma; HER2, human epidermal growth factor receptor 2; HL, Hodgkin lymphoma; HNSCC, head and neck squamous cell carcinoma; HR, hormone receptor; MCC, Merkel cell carcinoma; MET, mesenchymal-epithelial transition; MM, multiple myeloma; MRD, minimal residual disease; MSI-H, microsatellite instability-high; MTC, medullary thyroid cancer; NHL, non-Hodgkin lymphoma; NSCLC, non–small cell lung cancer; PD-L1, programmed death-ligand 1; Ph, Philadelphia chromosome; RA, regular approval; RCC, renal cell carcinoma; SEGA, subependymal giant cell astrocytoma; TNBC, Triple-Negative Breast Cancer; TSC, tuberous sclerosis complex; UC, urothelial carcinoma. | | | |

|  | | | | |
| --- | --- | --- | --- | --- |
| **Supplementary Table 3.** Accelerated Approvals for Malignant Hematology and Oncology Products That Were Withdrawn | | | | |
| **Product** | **Original Accelerated Approval Indication** | **Date of AA** | **Date of Withdrawal** | **Reason for Withdrawal** |
| Mobocertinib (Exkivity) | Refractory advanced NSCLC with EGFR exon 20 insertions | 9/15/2021 | 7/15/2024 | Lack of benefit |
| Infigratinib (Truseltiq) | Refractory advanced cholangiocarcinoma with FGFR2 gene fusions | 5/28/2021 | 5/16/2024 | Confirmatory clinical data not provided |
| Melphalan flufenamide (Pepaxto) | Refractory MM | 2/26/2021 | 2/23/2024 | Safety and efficacy concerns |
| Umbralisib (Ukoniq) | Refractory MZL | 2/5/2021 | 5/31/2022 | Safety |
| Umbralisib (Ukoniq) | Refractory FL | 2/5/2021 | 5/31/2022 | Safety |
| Pralsetinib (Gavreto) | Advanced or metastatic RET-mutant MTC | 12/1/2020 | 7/20/2023 | Confirmatory clinical data not provided |
| Belantamab mafodotin-blmf (Blenrep) | Refractory MM | 8/5/2020 | 2/6/2023 | Efficacy |
| Pembrolizumab (Keytruda) | Refractory metastatic SCLC | 6/17/2019 | 3/30/2021 | Lack of benefit |
| Atezolizumab (Tecentriq) | Newly diagnosed locally advanced or metastatic PD-L1+ TNBC | 3/8/2019 | 10/6/2021 | Lack of benefit |
| Duvelisib (Copiktra) | Refractory FL | 9/24/2018 | 12/17/2021 | Confirmatory clinical data not provided. |
| Nivolumab (Opdivo) | Refractory metastatic SCLC | 8/16/2018 | 12/29/2020 | Lack of benefit |
| Pembrolizumab (Keytruda) | Refractory locally advanced or metastatic PD-L1+ gastric or GEJ adenocarcinoma | 9/22/2017 | 2/4/2022 | Lack of benefit |
| Nivolumab (Opdivo) | Refractory HCC | 9/22/2017 | 7/23/2021 | Lack of benefit |
| Copanlisib (Aliqopa) | Refractory FL | 9/14/2017 | 3/18/2024 | Lack of benefit |
| Durvalumab (Imfinzi) | Refractory locally advanced or metastatic UC | 5/1/2017 | 2/19/2021 | Lack of benefit |
| Atezolizumab (Tecentriq) | Advanced or metastatic UC ineligible for cisplatin-based chemotherapy with PD-L1+ or ineligible for any platinum-based chemotherapy, regardless of PD-L1 | 4/17/2017 | 12/2/2022 | Lack of benefit |
| Ibrutinib (Imbruvica) | Refractory MZL | 1/18/2017 | 5/18/2023 | Efficacy |
| Olaratumab (Lartruvo) | Advanced soft tissue sarcoma | 10/19/2016 | 2/25/2020 | Lack of benefit |
| Atezolizumab (Tecentriq) | Refractory advanced UC | 5/18/2016 | 4/13/2021 | Lack of benefit |
| Panobinostat (Farydak) | Refractory MM | 2/23/2015 | 3/24/2022 | Confirmatory clinical data not provided |
| Idelalisib (Zydelig) | Refractory follicular B-cell NHL | 7/23/2014 | 2/18/2022 | Safety and efficacy concerns |
| Idelalisib (Zydelig) | Refractory SLL | 7/23/2014 | 2/18/2022 | Safety and efficacy concerns |
| Ibrutinib (Imbruvica) | Refractory MCL | 11/13/2013 | 5/18/2023 | Efficacy |
| Vincristine sulfate liposomal (Marqibo) | Refractory Ph- ALL | 8/9/2012 | 5/2/2022 | Confirmatory clinical data not provided |
| Romidepsin (Istodax) | Refractory peripheral T-cell lymphoma | 6/16/2011 | 7/30/2021 | Lack of benefit |
| Fludarabine phosphate (Oforta) | Refractory B-cell CLL | 12/18/2008 | 12/31/2011 | Confirmatory clinical data not provided |
| Bevacizumab (Avastin) | Newly diagnosed metastatic HER2- BC | 2/22/2008 | 11/18/2011 | Safety and efficacy concerns |
| Tositumomab and [¹³¹I] tositumomab (Bexxar) | Refractory low-grade follicular or transformed CD20+ NHL | 12/22/2004 | 10/23/2013 | Confirmatory clinical data not provided |
| Gefitinib (Iressa) | Refractory locally advanced or metastatic NSCLC | 5/5/2003 | 4/25/2012 | Lack of benefit |
| Gemtuzumab ozogamicin (Mylotarg) | Refractory CD33+ AML aged 60 years or older | 5/17/2000 | 11/28/2011 | Safety and efficacy concerns |
| Celecoxib (Celebrex) | To decrease adenomatous colorectal polyps in familial adenomatous polyposis patients. | 12/23/1999 | 6/8/2012 | Confirmatory clinical data not provided |
| Abbreviations: AA, accelerated approval; ALL, acute lymphocytic leukemia; AML, Acute Myeloid Leukemia; BC, breast cancer; CLL, chronic lymphocytic leukemia; FGFR2, fibroblast growth factor receptor 2; EGFR, epidermal growth factor receptor; FL, follicular lymphoma; HCC, hepatocellular carcinoma; HER2, human epidermal growth factor receptor 2; MCL, mantle cell lymphoma; MM, multiple myeloma; MZL, marginal zone lymphoma; MTC, medullary thyroid cancer; NHL, non-Hodgkin lymphoma; NSCLC, non–small cell lung cancer; PD-L1, programmed death-ligand 1; Ph, Philadelphia chromosome; RA, regular approval; SCLC, small cell lung cancer; SLL, small lymphocytic lymphoma; TNBC, Triple-Negative Breast Cancer; UC, urothelial carcinoma. | | | | |

| **Supplementary Table 4. Characteristics associated with Withdrawn Indications in the Univariable and Sensitivity Multivariable Logistic Regression Model ^a^** | | |
| --- | --- | --- |
| **Univariable Analysis** | |  |
| **Variable** | **OR (95% CI)** | ***P^b^*** |
| Initial approval (vs. supplemental) | 0·94 (0·41-2·14) | 0·88 |
| Orphan Drug Designation (vs. not) | 1·78 (0·73-4·37) | 0·20 |
| Priority Review designation (vs. not) | 0·79 (0·29-2·09) | 0·63 |
| Breakthrough Therapy Designation (vs. not) | 0·28 (0·10-0·75) | 0·012 |
| One trial supporting accelerated approval (vs. multiple trials) | 1·60 (0·55-4·62) | 0·39 |
| Hematologic (vs. solid) | 2·32 (1·07-5·26) | 0·043 |
| Genome-targeted therapies (vs. non-genome targeted therapies) | 0·26 (0·08-0·80) | 0·019 |
| Immune checkpoint inhibitor (vs. non-immune checkpoint inhibitor) | 1·34 (0·53-3·42) | 0·54 |
| Confirmatory study underway at the time of accelerated approval | 0·41 (0·17-0·98) | 0·044 |
| Number of patients | 1·01 (0·99-1·01) | 0·18 |
| Randomized controlled trial (vs. single-arm trial) | 0·83 (0·36-1·94) | 0·67 |
| Phase I-II (vs. phase III) | 2·87 (0·80-10·28) | 0·11 |
| Time to event endpoint (vs. non-time to event endpoint) | 0·83 (0·25-2·69) | 0·75 |
| ESMO-MCBS Low clinical benefit (vs. intermediate and high benefit) | 4·83 (1·56-14.88) | 0·006 |
| **Multivariable Analysis** | | |
| **Variable** | **OR (95% CI)** | ***P^c^*** |
| Breakthrough Therapy Designation (vs. not) | 0·28 (0·10-0·75) | 0·012 |
| Confirmatory study underway at the time of accelerated approval^d^ | 0·30 (0·07-1·28) | 0·10 |
| Hematologic (vs. Solid) | 1·15 (0·40-3·97) | 0·70 |
| Genome-targeted therapies (vs. non-genome targeted therapies) | 0·37 (0·09-1·52) | 0·17 |
| ESMO-MCBS Low clinical benefit (vs. intermediate and high benefit) | 4·83 (1·56-14·88) | 0·006 |
| ^a^ This analysis included 133 accelerated approvals  ^b^  Based on univariable logistic regression. *P* values are two-sided, with significance set at p < 0·05.  ^c^ Based on multivariable logistic regression. A sensitivity analysis was performed by repeating the multivariable regression, including only variables significant in the univariate analysis. Significance was initially set at p < 0·05 and adjusted using Bonferroni correction for multiple comparisons.  ^d^ Indications with confirmatory trials underway at the time of accelerated approval were initially associated with lower odds of withdrawal (OR 0·21; 95% CI, 0·06-0·81; p=0·02), but this lost significance after Bonferroni correction. | | |
